# The History of the Role of Urologists in the Spinal Units in the United Kingdom

**DOI:** 10.5812/numonthly.3427

**Published:** 2012-09-24

**Authors:** John Russell Silver

**Affiliations:** 1 National Spinal Injuries Centre, Stoke Mandeville, Buckinghamshire, UK

**Keywords:** Urology, Spinal Injuries

Spinal injuries have been known since antiquity and until the 20th century, carried an inevitable fatal prognosis. Patients died either immediately from their intercurrent injuries and acute fulminating urinary tract infections, or they died in the following weeks from chronic pyelonephritis and pressure sores. Any treatment was palliative, easing the patient’s pathway to the grave.

Today, as a result of pioneering work started prior to the Second World War by Donald Munro in the United States and during the Second World War by Ludwig Guttmann in the United Kingdom, the situation has changed remarkably, provided the patient with an acute spinal injury is admitted to a specialised centre where all the facilities to investigate and treat the patient comprehensively are available (1). Dedicated medical staff, comprising of a neurosurgeon, an orthopaedic surgeon, a plastic surgeon, a urological surgeon, a physical medicine consultant, and a radiologist, should work as a team with one consultant taking the lead and assuming overall responsibility for the patients. Provided there is appropriate radiological backup with CT and MRI scanners and the support of the intensive care unit with devoted occupational and physiotherapy staff, and on discharge, follow up is maintained under the care of the unit, the patient can expect a near normal life expectancy and return to gainful employment (1).

It is apparent that the management of the paralysed bladder is paramount to the successful management of the patient. How this evolved is of particular interest and there are many lessons to be learned. Prior to the first World War, traumatic spinal injuries were rare. It took Hulke 24 years to accumulate data on 33 patients, 22 of whom were under his care (2). Warfare provided a unique series of casualties but there were no survivors from the Crimean War and 75% of the spinal injury patients From the Boer War died within a few weeks (2, 3). The First World War was a conflict of unprecedented proportions with 1,662,625 casualties seen in the British Army alone and the majority of paraplegics died soon after injury. Of those who survived long enough to reach a base hospital in France, Vellacot and Webb Johnson recorded 66 patients with spinal injuries, 21 of whom died, 8 from renal failure. Of the 339 patients admitted to the King George V Hospital, 160 died of urinary tract infection (4). The method of managing the bladder was by means of an indwelling catheter or by intermittent catheterisation. The way this was peformed produced disastrous effects and was largely responsible for the high mortality. Thompson Walker, the sole urologist at the Royal Star and Garter Home, where chronic patients were admitted, stated that management of the bladder in the First World War was the surgical failure of the War (5, 6).

Little changed between the Wars and when the Second World War broke out, a committee was formed under the chairmanship of George Riddoch who had treated the patients during the First World War at the Empire Hospital. In 1940, they opened a series of acute units with neurosurgical, orthopaedic, operative and pathology support but interestingly no urological consultants. The units were just as bad as during the First World War, with patients staying in for 3 years and being no better after this time than when first admitted (1).

In view of the late arrival of urological management for these patients, it is interested to study how the urological care came to be instituted within the spinal units in the United Kingdom. The author has unique experience of this, having started practising spinal medicine in 1956.

The reasons for this lack of urological care were twofold: there was a shortage of urologists in the United Kingdom and there was a mistaken attitude towards the role of urologists. Doctors were of the view that they should only call in the urologist when all other treatment had failed, consequently, the urologist was confronted with a desperate situation and could only function on a salvage basis. At Stoke Mandeville, when the unit was opened in 1944, Eric Riches was the visiting urological surgeon but he was only asked to see patients by Sir Ludwig Guttmann, the autocratic director of the unit, when all other treatment had failed, and latterly, he was not called into the unit at all.

At Winwick, there was no urological input and the unit eventually closed down and was reopened at the instigation of Charles Wells, professor of surgery at Liverpool, at Southport in 1948, with a visiting urologist, Cosby Ross and a neurosurgeon in charge of the unit. Very rapidly, the importance of urology was recognised and a young surgeon in training, Norman Gibbon elected to do his Mastership thesis on the management of the bladder in spinal patients. He used to cycle to the unit from the Southport railway station carrying his urology manometers in one hand. He carried out urological research upon these patients and subsequently became the visiting consultant, doing pioneering research on the management of the bladder, particularly external sphincterotomy and outflow surgery and the development of the Gibbon catheter. When I was appointed in 1965, I met him and he explained to me that he saw half the patients one week and the other half the week after. We carried out collaborative rounds and he was involved in every stage of the treatment. We wrote a number of papers together on the management of the bladder. When I returned to Stoke Mandeville in 1970, I carried on the lessons I had learned in Southport and instituted a combined ward round with the bacteriologist and the visiting urologist Griffiths Fellows, who devoted nearly all his time to seeing my patients. Fellows saw my patients on a regular basis from admission to their discharge and followed them up. We wrote several papers together and if I was absent, the treatment would carry on without interruption. There was no need for me to dictate the treatment.

The situation in Sheffield was similar. The urology was being carried out by John Williams on a visiting basis. His senior registrar (David Gwyn Thomas) in urology at that time embarked on a full-time research project – setting up a combined urodynamic/radiological screening service on the spinal unit. This led to his appointment as Consultant Urologist to the spinal injuries unit in 1974. As well as his urological work he shared in the general management of the patients, including the emergency care of the acutely injured patient. The continuing research interest over the next 30 years led to an increased understanding of the neurogenic bladder and has resulted in the appointment of several consultant urologists with a greater input on spinal injury units in the United Kingdom and overseas.

These three committed urologists worked as equal members of a team. They have been fully fledged members of the spinal injury team, they have been backed up by a fully staffed urodynamic, radiology and operating sessions incorporated within the spinal unit, and they have been involved from the outset in the management of the patients and the follow up of the patients. Patients, who are discharged from the spinal unit after discussion with the treating staff, should be discharged with a treatment to meet their requirements, with bladders that they can manage themselves. They should be followed up regularly at the spinal centre. It is no good sending a patient off to a satellite urology service at some further date where the skills of the spinal unit are not available.

The situation is entirely comparable to the management of patients with malignant disease when the oncologist, the surgeon, the physician and the support staff all work together as a committed team on an equal basis to discuss the best management of the patient.
